# Pathogenesis and Treatment of Cytokine Storm Induced by Infectious Diseases

**DOI:** 10.3390/ijms222313009

**Published:** 2021-11-30

**Authors:** Xi-Dian Tang, Tian-Tian Ji, Jia-Rui Dong, Hao Feng, Feng-Qiang Chen, Xi Chen, Hui-Ying Zhao, De-Kun Chen, Wen-Tao Ma

**Affiliations:** College of Veterinary Medicine, Northwest A&F University, Yangling District, Xianyang 712100, China; tangxidian@nwafu.edu.cn (X.-D.T.); jtt@nwafu.edu.cn (T.-T.J.); dongjiarui@nwafu.edu.cn (J.-R.D.); 99fen98hao1900@nwafu.edu.cn (H.F.); 18724391203@nwafu.edu.cn (F.-Q.C.); chenxi2017@nwafu.edu.cn (X.C.); zhaohuiying@nwafu.edu.cn (H.-Y.Z.)

**Keywords:** cytokine storm, inflammation, infectious disease, pathophysiological mechanism, treatment strategies

## Abstract

Cytokine storm is a phenomenon characterized by strong elevated circulating cytokines that most often occur after an overreactive immune system is activated by an acute systemic infection. A variety of cells participate in cytokine storm induction and progression, with profiles of cytokines released during cytokine storm varying from disease to disease. This review focuses on pathophysiological mechanisms underlying cytokine storm induction and progression induced by pathogenic invasive infectious diseases. Strategies for targeted treatment of various types of infection-induced cytokine storms are described from both host and pathogen perspectives. In summary, current studies indicate that cytokine storm-targeted therapies can effectively alleviate tissue damage while promoting the clearance of invading pathogens. Based on this premise, “multi-omics” immune system profiling should facilitate the development of more effective therapeutic strategies to alleviate cytokine storms caused by various diseases.

## 1. Introduction

### 1.1. The Definition of Cytokine Storm

**Figure 1:**
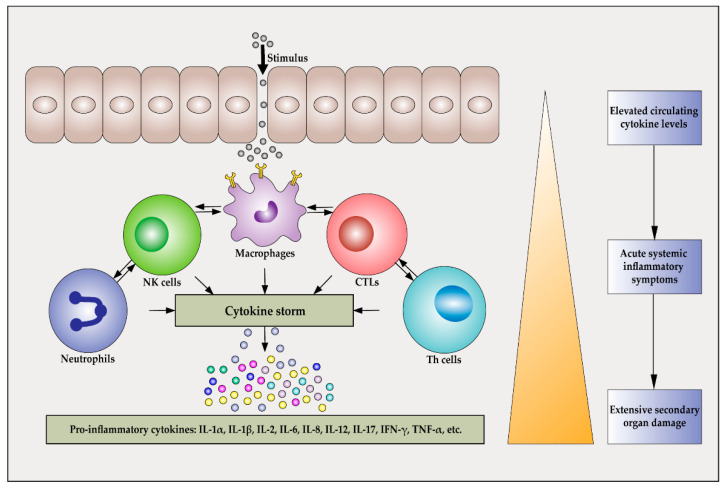
Cells that can be involved in a cytokine storm. Various types of cells, such as neutrophils, natural killer (NK) cells, macrophages, cytotoxic T lymphocytes (CTLs), and T helper cells (Th cells), are intimately involved in the initiation and progression of cytokine storms. These cells interact with each other and can influence each other’s activities.

Cytokine storm, also referred to as cytokine release syndrome (CRS) or hyper-cytokine syndrome [1], is back in the spotlight due to its association with the coronavirus pandemic of 2020. However, the concept of cytokine storm is not limited to complications of coronavirus disease 2019 (COVID-19) [2,3], but has been observed during diverse disease outbreaks, including the severe acute respiratory syndrome (SARS) outbreak in 2003 and the swine influenza outbreak in 2009. The term cytokine storm was first used in the literature in 1993 by Ferrara et al. to describe the pathogenic side effect of graft-versus-host disease, a transplant syndrome, following allogeneic hematopoietic stem cell transplantation [4,5]. The concept of cytokine storm has been widely used in infectious diseases since the outbreak of H5N1 influenza infection in early 2000 when it was used to describe the excessive production of inflammatory cytokines following infection [6]. In the case of cancer treatment, cytokine storm was first used in 2010 to describe the side effects of chimeric antigen receptor T-cell immunotherapy (CAR-T) cell therapy [7,8]. At this historical stage, cytokine storm was considered to be an influenza-like syndrome that occurred after systemic infection and immunotherapy [9]. For example, Yersinia pestis infection has previously caused pandemics and triggered excess cytokine production in alveolar macrophages, causing cytokine storms [10]. After 2010, studies on cytokine storms gradually increased, but the definition of cytokine storms is still different. In 2012, Tisoncik et al. proposed that cytokine storm is a harmful inflammatory disorder rather than a beneficial host response [11]. In 2016, Liu et al. proposed that “cytokine storm” is caused by an inflammatory response induced by an uncontrolled immune system, the term “storm” describes its pathogenesis. Additionally, “cytokine storm” is a condition characterized by the excessive pro-inflammatory response and inadequate anti-inflammatory response [12]. In the period 2017–2018, Teijaro et al., J.R. et al. and Shimabukuro-Vornhagen, A. et al., respectively, used this term to describe the abnormal production of soluble substances during severe pathogen infection [13] and to describe systemic inflammatory responses caused by multiple factors, including infection [14]. In 2020, David C. Fajgenbaum and Carl H. systematically described the definition of cytokine storm, which, in their opinion, is a condition associated with increased circulating cytokine levels, systemic inflammatory clinical symptoms and severe secondary organ damage [1] (Figure 1).

### 1.2. Cytokines Associated with Cytokine Storm

Pathogen-induced infections generally cause rapid and massive production of various cytokines, such as tumor necrosis factor (TNF)-α, interleukin (IL)-1, IL-6, IL-12, interferon (IFN)-α, IFN-β, IFN-γ, monocyte chemoattractant protein-1 (MCP-1) and IL-8 [15,16], which can promote the development of harmful inflammation. If cytokine storm occurs, the body’s immune system will respond to the invaders by producing excessive quantities of inflammatory cytokines [17]. This high-level release of cytokines generally promotes downstream processes that can damage multiple tissues and organs [14,17].

It is worth noting that IL-6 is an immune-regulatory factor that can play either pro-inflammatory or anti-inflammatory roles depending on existing conditions [18,19,20]. The pro-inflammatory role of IL-6 depends on the binding of IL-6 to its soluble receptor to initiate monocytes differentiation into macrophages, leading to recruitment of other immune cells to inflammatory sites, inhibition of regulatory T (Treg) cell activities, and triggering of acute immune-pathological reactions [3]. Importantly, IL-6 may serve as a biomarker for cytokine storm disease severity and prognosis [15,21,22,23].

The basis of CRS is rooted in hypersensitive immune system activation involving a variety of cells that culminates in the release of large amounts of cytokines, with cytokine profiles varying from disease to disease [8]. For example, cytokines involved in CAR-T therapy-triggered CRS include IFN-γ, IL-2, IL-2Rα, IL-6, sIL-6R, granulocyte-macrophage colony-stimulating factor (GM-CSF), IL-1, IL-10, IL-12, tumor necrosis factor-α (TNF-α), IFN-α, MCP-1, and macrophage inflammatory protein-1α (MIP-1α) [8,24,25]. By contrast, CRS caused by SARS mainly leads to a release of IL-1β, IL-6, IL-12, IFN-γ, and MCP-1, while the cytokine storm caused by Middle East respiratory syndrome (MERS) coronavirus has mainly involved the release of IFN-γ, TNF-α, IL-15, and IL-17 [26,27,28]. As another example, monitoring of COVID-19-associated CRS patient cytokine profiles in 2019 revealed higher levels of IL-2, IL-7, IL-10, G-SCF, MCP-1, MIP-1α, and TNF-α in plasma of intensive care unit (ICU) patients versus plasma of non-ICU patients [16]. The following table summarizes cytokines that are significantly elevated in patients afflicted with common diseases that trigger cytokine storms (Table 1).

### 1.3. The Clinical Effects, Therapeutic Approaches, and Prognosis of Cytokine Storms

The clinical characteristics of cytokine storms are systemic inflammation and multi-organ dysfunctions, and even systemic organ failure [16,29]. Almost all patients with cytokine storms experience fever [30]. In addition, patients may experience fatigue, anorexia, headache, diarrhea, myalgia, and neurological symptoms [16,31]. These symptoms may be directly caused by cytokine-induced tissue damage or physiological changes in the acute phase, or they may result from immune cell-mediated responses [1]. The clinical features of the patients will rapidly develop into diffuse intravascular coagulation, accompanied by vascular obstruction or massive bleeding, dyspnea, hypoxemia, hypotension, hemostatic imbalance, and vasodilatory shock [16,32].

The general treatment strategy for cytokine storms involves supportive care to maintain organ function, control of the underlying disease and elimination of triggers for abnormal immune system activation and targeted immunomodulation to limit the collateral damage of the activated immune system [33]. In addition to these conventional treatments, there are many alternative therapeutic approaches for cytokine storms. For example, for the treatment of SARS-CoV-2 infection, resveratrol, vitamin D, and melatonin were found to improve the body’s immune system and have been suggested to serve as potential anti-SARS-CoV-2 inhibitor molecules [34,35,36]. It has also been found that natural products, particularly plant ingredients, are unique sources of a variety of effective and novel medicines for CRS. Immune stimulants such as vitamins, iron, zinc, monochrome, caffeic acid, and gallic acid can activate the immune system’s defense mechanisms and become powerful weapons in the fight against COVID-19 [36,37,38]. In addition to complementary therapy, combination therapy of steroids and tocilizumab, an IL-6 receptor antagonist, may be a safe and effective treatment for cytokine storms associated with COVID-19 [20,22,39].

There are several potential prognosis indicators for cytokine storms [40]. For example, several studies showed that lymphocytopenia was associated with more severe cases of COVID-19 [16,31,41,42]. In addition, increased neutrophil to lymphocyte ratio and high platelet to lymphocyte ratio may indicate more serious cytokine storms for prolonged hospitalization [41]. Biomarkers such as high serum procalcitonin and ferritin are also associated with adverse prognostic factors [43].

## 2. Pathophysiological Mechanisms of Cytokine Storms

Pathophysiological mechanisms underlying cytokine storms are unknown. However, the results of previous studies suggest that CRS occurrence is linked to an imbalance between pro-inflammatory and anti-inflammatory mechanisms resulting from effects of various intercellular cytokine interactions and regulatory disorders [14]. Normally, healthy hosts possess a wide range of regulatory mechanisms to control inflammatory responses. For example, during pathogen infection, Treg cell activity is increased, immunosuppressive cytokines are produced, and inflammatory responses are suppressed [44,45,46]. Meanwhile, regulatory B cells also play an immunomodulatory role in pathogen infection, with this type of cell inducing immunosuppression through the production of cytokines IL-10 and IL-35 [47,48,49]. Nevertheless, when the host pro-inflammatory response overwhelms host immunoregulatory cell responses, cytokine storms may result. Underlying pathophysiological mechanisms for imbalances between pro-inflammatory and anti-inflammatory mechanisms include inflammatory cell infiltration with inhibition of regulatory cell function, as well as high-level expression of pro-inflammatory cytokines that overwhelms low-level expression of anti-inflammatory cytokines [50,51]. For example, levels of various pro-inflammatory cytokines in serum and bronchoalveolar lavage fluid samples obtained from cystic fibrosis patients were significantly greater than corresponding levels in controls [50,52]. Due to the predominantly pro-inflammatory response in cystic fibrosis patients, more and more neutrophils are induced to migrate to the lungs, where the resulting neutrophilic infiltration induces lung damage. In addition, cystic fibrosis patients have been shown to produce decreased levels of anti-inflammatory cytokines, while their Treg cells exhibited lower activity than Treg cells of healthy controls [50,52]. Based on the fact that CRS involves cytokine release, researchers generally agree that immune cells and other types of cells drive CRS-associated processes. These cells function within a complex regulatory network consisting of lymphocytes, macrophages, dendritic cells, monocytes, and endothelial cells that normally regulates cell secretion of various cytokines, such as IFN-γ, TNF-α, and IL-6 but under certain conditions supports CRS development [53,54]. Clues to underlying cellular mechanisms that drive CRS development include observations of the following CRS-associated cellular changes: T helper cell 17 (Th17) cells and T follicular helper (Tfh) cells differentiate, CD8^+^ cytotoxic T cells and B cells are activated and differentiation and development of Treg cells are suppressed [3,55,56].

Many factors can trigger CRS, including various therapies, pathogen-induced triggers (e.g., bacterial sepsis, influenza virus, and COVID-19), autoimmune conditions (e.g., autoinflammatory disorders), monogenic disorders (e.g., primary or secondary hemophagocytic lymphohistiocytosis), and iatrogenic interventions (e.g., CAR T-cell therapy, blinatumomab, other T-cell-engaging immunotherapies, and gene therapies) [1,25,57]. In the present review, we focus on pathogen-induced CRS and the underlying mechanisms involved in CRS generation. CRS caused by pathogen infection is classified and summarized in terms of the type of pathogen, the amount of infection, and the level of growth of pathogens.

### 2.1. The Type of Pathogen

#### 2.1.1. The Pathophysiological Mechanism of CRS Caused by Influenza Virus Infection

Influenza A virus has been classified into many different subtypes and which have been observed to be transmitted among animals and humans [58,59,60,61]. During an outbreak of the H5N1 influenza virus in humans in Southeast Asia, disease morbidity and mortality were linked to CRS as a key factor associated with influenza virus pathogenesis [6]. Intriguingly, the blockade of cytokine storms was shown to provide greater protection against morbidity and mortality than the protection provided by antiviral therapy [62].

**Figure 2:**
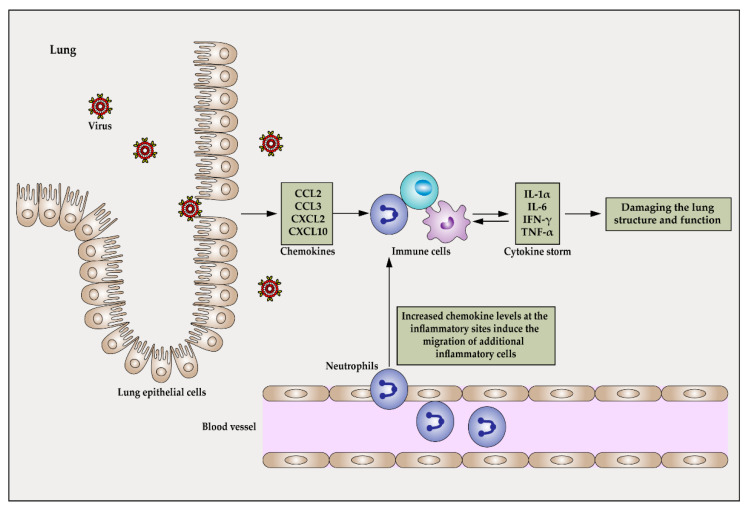
Cytokine storm in the lung after influenza virus infection. Influenza virus is mainly transmitted through the respiratory tract, such as the trachea, into the alveoli. Once inside the body, the virus can also be transmitted through blood circulation. When the influenza virus infects lung epithelial cells and alveolar macrophages, it replicates, causing a release of a large number of viruses that induce the release of host cytokines. Activation of macrophages by cytokines and chemokines leads to additional immune responses that can trigger a cytokine storm. At the same time, increased chemokine levels at the inflammatory site induce migration of additional cytokine-releasing inflammatory cells to the site, thus amplifying the cytokine storm effect.

Influenza virus infection is accompanied by the production of a variety of cytokines, including IFN-γ, IL-1α, IL-6, and chemokines CCL2, CCL3, CXCL2, and CXCL10 [63]. IL-1α, IL-6, and TNF-α perform a variety of functions that may influence influenza virus pathogenesis [59,63]. Meanwhile, secretion of CCL2, CCL3, CXCL2, and CXCL10 is known to cause large numbers of innate immune cells to enter lung tissue [64], where they release more and more cytokines and amplify the CRS process, thereby damaging the basic lung structure and preventing proper lung function [65,66] (Figure 2).

Once in the lungs, innate immune cells produce excessive quantities of pro-inflammatory cytokines and chemokines [63,64,67]. Traditionally, infiltration of lung epithelium by inflammatory cells has been thought to be the primary cause of CRS. However, several studies have shown that pulmonary endothelial cells can also participate in severe CRS development and thus should be targeted by therapies designed to suppress excessive innate inflammatory responses within the lung [65,68].

In the case of influenza virus-induced CRS, it has been suggested that immune-regulatory therapy may improve therapeutic outcomes when used alone or in combination with antiviral drugs [12]. Based on this suggestion, current strategies for treating this type of CRS include corticosteroids, peroxisome proliferator-activated receptor agonists, sphingosine-1-phosphate receptor 1 agonists, cyclooxygenase-2 inhibitors, antioxidants, anti-TNF therapy, intravenous immunoglobulin therapy, and other therapeutic strategies [12,69,70,71].

#### 2.1.2. The Pathophysiological Mechanism of Bacterial Sepsis-Induced CRS

Along with influenza, bacterial sepsis has also been associated with severe CRS [72,73,74]. Sepsis is a severe clinical syndrome whereby the host cannot control the spread of pathogens within the body [75,76]. Cytokine storms resulting from either endotoxin or exotoxin-induced cytokine overproduction can lead to harmful effects (e.g., sepsis, toxic shock syndrome) [77,78]. Sepsis has a mortality rate of nearly 20% and thus is a major public health problem. Both Gram-positive and Gram-negative bacteria can cause sepsis. Specifically, common Gram-positive bacterial pathogens include *Staphylococcus aureus* and *Streptococcus pneumoniae*, while Gram-negative pathogens include *Escherichia coli*, *Klebsiella spp*., and *Pseudomonas aeruginosa* [79]. Unfortunately, all clinical trials of treatments designed to suppress sepsis-associated inflammatory responses or CRS have failed, warranting additional studies to enhance our understanding of CRS-induced sepsis [75].

**Figure 3:**
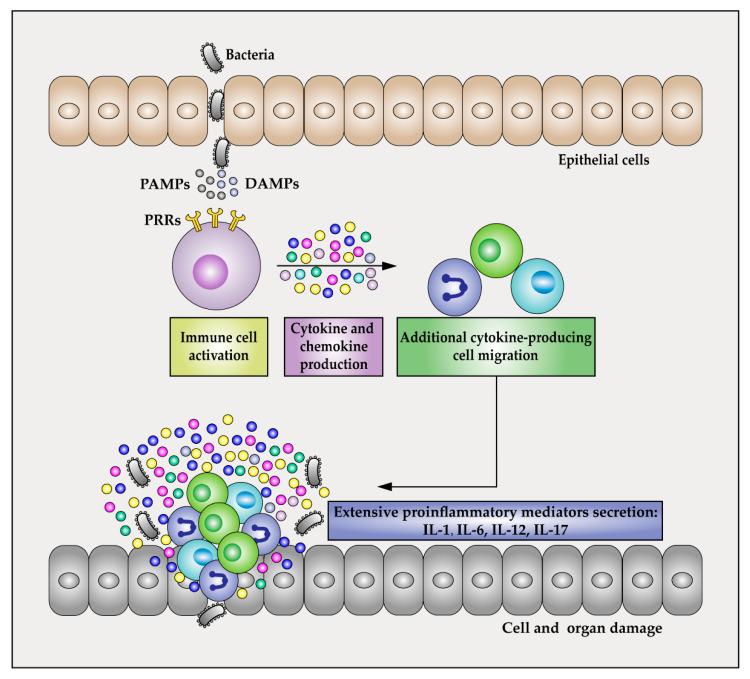
Cytokine storms during sepsis. When microorganisms invade the body, activation of innate immunity is initiated by recognition and binding of pattern recognition receptors (PRRs) to pathogen-associated molecular patterns (PAMPs) or damage-associated molecular patterns (DAMPs) followed by triggering of a series of activation or phosphorylation reactions that induce an inflammatory response. Many pro-inflammatory cytokines have been studied during sepsis-induced cytokine storms, including IL-1, IL-6, IL-12, and IL-17. Sepsis-induced cytokine storm leads to activation and recruitment of leukocytes that promote excessive inflammation that seriously damages cells and organs, often leading to multi-organ failure and death.

When bacteria and other microorganisms invade the body, they are detected by the innate immune system. Activation of innate immunity involves triggering pattern recognition receptors (PRRs) which recognize pathogen-associated molecular patterns (PAMPs) or damage-associated molecular patterns (DAMPs) [80]. PRRs are widely expressed in a variety of innate immune cells such as macrophages, monocytes, and dendritic cells [81]. During infection by invading pathogens, the recognition of PAMPs by different PRRs transmit the signal of infection and contribute to the first step in the development of an effective innate immune response against pathogens and to induce sepsis [82,83]. For example, when bacterial PAMPs such as lipopolysaccharide, lipoteichoic acid, peptidoglycan, and CpG-DNA were recognized by corresponding TLRs (TLR4, TLR2, TLR5, TLR9, etc.), myeloid differentiation primary response protein 88 (MyD88)-dependent and MyD88-independent signaling pathways will be activated [84]. The corresponding adaptor molecules such as IL-1 receptor-associated kinase 1 (RAK-1) and tumor necrosis factor receptor-associated factor 6 (TRAF6) are phosphorylated and formed a complex to induce the activation of nuclear factor kappa-B (NF-κB), further inducing the secretion of a variety of pro-inflammatory cytokines [84,85,86]. Many pro-inflammatory cytokines, including IL-1, IL-6, IL-12, and IL-17, play critical roles during early sepsis [73,87]. By contrast, different cytokines participate in sepsis progression, with increased levels of IL-1β, IL-6, IL-8, IL-12, IFN-γ, granulocyte colony-stimulating factor, and TNF-α observed in non-survivors as compared to survivors [87,88,89]. In particular, TNF-α, G-CSF, and chemokines, normally key players in host responses to infection, are expressed at high levels in septic patients [75], where they trigger excessive inflammation that seriously damages cells and organs, and can ultimately lead to multi-organ failure and death [74,75] (Figure 3).

Treatments that have been shown to alleviate CRS can provide clues to mechanisms involved in CRS initiation and progression while also revealing potential therapeutic targets [76]. One effective CRS treatment, anakinra, is a recombinant human IL-1 receptor antagonist that has been shown in previous studies to be safe and effective for alleviating CRS, thus confirming that IL-1 is a central player in severe sepsis-associated cytokine storms and a potential therapeutic target [90,91]. Meanwhile, results of other studies have suggested that targeting G protein-coupled receptor 174 can alleviate CRS, providing another clue to mechanisms underlying sepsis-induced CRS [92]. This receptor plays an important role in the initiation of sepsis by regulating macrophage polarization and pro-and anti-inflammatory cytokine secretion such that targeting these processes might alleviate sepsis-induced CRS [92]. As another mechanistic clue to CRS pathogenesis, injections of Chinese herbal medicine Xuebijing have been shown to prevent cytokine storms and improve the survival of septic shock patients [93]. Mechanistically, Xuebijing treatment partially inhibited inflammation by regulating the balance between Tregs and Th17 cells [93]. Interestingly, Karbian and colleagues found that apoptotic cell administration can efficiently reduce the severity of sepsis-induced cytokine storm in cecal ligation and puncture mouse models [80,94].

#### 2.1.3. The Pathophysiological Mechanism of CRS Caused by SARS-CoV-2 Infection

SARS-CoV-2 is a highly pathogenic and infectious coronavirus that causes the COVID-19 pandemic. Increasing evidence has suggested that cytokine storm is closely associated with the severity of COVID-19 [95]. COVID-19-related CRS patients exhibited increased plasma levels of IL-2, IL-7, IL-10, G-SCF, MCP-1, MIP-1α, and TNF-α [16]. Specifically, ICU patients showed significantly higher levels of those pro-inflammatory cytokines compared with non-ICU patients [16]. In addition, increased IL-6 levels were found to be associated with decreased survival time of COVID-19 patients, and IL-6 level was suggested to serve as a biomarker of CRS severity [22,23,96]. Consistent with these findings, IL-6 signaling blockade alleviated the clinical symptoms immediately in severe COVID-19 patients and has been recommended for the treatment of severe COVID-19 patients [97,98,99]. In addition, several findings have shown that IL-1 receptor antagonist treatment was therapeutic in COVID-19 patients and improved the overall survival of these patients [100,101,102]. Furthermore, the level of inflammatory cytokines can be reduced by inhibiting the Janus kinases (JAKs)-associated signaling pathways [103,104]. In addition, therapeutic plasma exchange has also shown potential in treating severe COVID-19 by reducing injurious cytokines [105,106,107].

### 2.2. The Amount of Pathogen Infection and the Level of Pathogen Growth

In general, there is a positive correlation between the number of pathogenic organisms present during pathogen exposure and the probability that a person exposed to those organisms becomes sick [108]. In addition, the pathogen reproduction rate in the host is another prerequisite determinant of whether pathogenic microorganisms induce illness [109]. Although viruses and bacteria are commonly recognized by the host immune system through pattern recognition receptors such as Toll-like receptors (TLRs), different mechanisms are required in response to their infections.

In response to viral infections, TLR3, TLR7, TLR8, or TLR9 are usually necessary for antigen recognition and pro-inflammatory cytokine production [110,111]. For example, CpG content in viruses constitutes an important prerequisite for the activation of TLR9 signaling [112]. In SARS-CoV and SARS-CoV-2, two highly pathogenic coronaviruses, CpG content is much higher than that in other coronaviruses isolated from humans, making them highly efficient in eliciting severe pro-inflammatory immune responses and tissue damage [110,112].

In addition, severe pneumonia caused by coronaviruses is usually associated with rapid viral replication, a large amount of inflammatory cell infiltration, and the generation of an intense pro-inflammatory cytokine/chemokine response. These observations indicate that viral load during initial patient exposure and viral replication levels in the host are closely and positively correlated with the degree of disease progression and subsequent CRS development [27]. During COVID-19 disease, the viral load in the nasopharynx of a patient is closely related to cytokine levels. In mild cases, the viral load in the nasopharynx decreased, while in severe cases, the viral load in the nasopharynx increased and there was a cytokine storm [113]. A variety of inflammatory cytokines were abnormally expressed and homeostasis was broken, suggesting that the immune response of the body was closely related to cytokine levels and viral load. Additionally, the viral load is reflected by the amount of virus infections and the level of virus growth and reproduction [1].

During bacterial infections, the recognition of bacterial PAMPs such as lipopolysaccharide, lipoteichoic acid, peptidoglycan, porins, flagellin, and CpG-DNA by their corresponding TLRs (TLR4, TLR2, TLR5, and TLR9) is important in inducing the synthesis and release of pro-inflammatory cytokines and chemokines [82,114,115]. For example, in cecal-ligation and puncture (CLP)-induced sepsis mice, deletion of TLR4 or TLR2 resulted in significantly decreased levels of pro-inflammatory cytokines (i.e., IL-1β, TNF-α, IL-6, and IL-8) and preserved tissue damages [114]. Additionally, the expression of specific TLRs is usually upregulated in response to severe bacterial infections and this could further aggravate the pro-inflammatory immune response and even the cytokine storm, a phenomenon commonly observed during sepsis [82,114].

## 3. Treatment Strategies for CRS Induced by Infectious Diseases

In short, alleviation of CRS using immunomodulatory approaches and addressing the root cause of CRS are both needed to cure patients with CRS [57]. Therapeutic options include steroids, intravenous immunoglobulins, inhibitors of JAKs, and selective cytokine blockade treatments, such as anakinra, an IL-1 receptor antagonist, or tocilizumab, an IL-6 receptor antagonist [22,116,117,118]. During the progression of infectious disease, general treatments for alleviating harmful CRS effects on the body of the host include the following: basic supportive therapy to maintain key organ functions; medications that address root causes of CRS-triggering diseases while eliminating other abnormal immune system activation triggers; and targeted immune dysregulation or non-specific immune suppression to limit collateral damage resulting from inappropriately excessive immune system activation [119]. In actual practice, clinically administered treatments include antibiotics, antiviral drugs, bacteriophages, and monoclonal antibodies [120,121].

### 3.1. Targeting the Pathogen

Current treatment strategies for alleviating pathogen-induced CRS include antibiotics, antiviral drugs, bacteriophages, and monoclonal antibodies [122]. Antibiotics were discovered in the 20th century, and continue to be used frequently to eliminate bacterial causes of numerous infectious diseases [123,124]. However, due to selective pressures associated with antibiotic use and misuse, drug resistance has emerged rapidly [125]. As for antiviral drugs, far fewer approved antiviral treatments are available as compared to antibiotics. Currently available antiviral drugs are highly specific, targeting only a few virus species, and many of them have side effects [122].

In cases when effective and promising antibiotics and antiviral drugs do not exist, bacteriophage-based treatments have been used and are attracting more and more attention as viable treatments [121]. Although bacteriophages reproduce easily and have few side effects, several distinct bacteriophages targeting the same bacterial species must be delivered as a mixture to prevent the emergence of resistance [126]. Due to the high degree of specificity of bacteriophage-based treatments, an accurate diagnosis must be made to select appropriate bacteriophage mixtures for combating each particular type of infection [126].

In recent years, monoclonal antibody (mAb)-based disease treatments have gained wide acceptance as CRS treatments [22,120], due to their outstanding specificity and minimal adverse side effects [122]. At present, mAbs are commonly used as targeted therapies to treat infectious diseases, transplant rejection, and autoimmune diseases. However, the use of mAbs is also associated with several risks, such as acute allergic reactions and serum diseases, which must be reduced to improve drug safety [127].

### 3.2. Targeting the Host

During the progression of serious infectious diseases, general treatment strategies used to calm CRS include anti-shock therapy and supportive measures to maintain blood volume, water content, and electrolyte balance (e.g., transfusions, mechanical ventilation, and routine infusions). In addition, many additional targeted therapies are now being used to treat CRS.

One new type of anti-CRS therapy, hormone therapy, is used clinically to inhibit both immune cell activation and cytokine production. Corticosteroids, a type of steroid hormone, exert an anti-inflammatory effect while also regulating the transcription of anti-inflammatory genes [119,128]. Therefore, corticosteroids have been widely used as anti-inflammatory treatments, with specific types of corticosteroids used to treat different syndromes [12,128]. For example, methylprednisolone is used to treat most rheumatic illnesses, while dexamethasone is often used to treat familial hemophagocytic lymphohistiocytosis [57]. Nevertheless, patients taking corticosteroids must be monitored for adverse side effects, warranting research to find alternative treatments for inflammatory disorders.

CRS progression is accompanied by significant and unusual increases in levels of various cytokines, some of which play important roles in inflammatory and pathological processes. Thus, neutralization of abnormally elevated cytokines in the body through the administration of specific monoclonal antibodies may reduce inflammation [22,119,120]. For example, IL-1 receptor blockade has been associated with reduced mortality of sepsis patients [91,118], while significant alleviation of CRS associated with excessive IL-6 synthesis has been achieved using the humanized anti-IL-6 receptor antibody tocilizumab, which blocks IL-6 synthesis [18,22]. However, neutralization of a particular cytokine present at high levels in circulation is not always achievable using any single agent. By contrast, blocking a cytokine with a low or normal circulating concentration is effective if the cytokine is a key component of the hyper-inflammatory circuit or if it is likely to present at high levels in tissues [1,100,102]. About other potential strategies, stimulation of key factors within anti-inflammatory pathways, targeting overactive immune responses, and manipulation of regulatory T cells all hold promise [122]. For example, patients with COVID-19 may have elevated cytokines such as IL-6, IL-2, IL-7, and IL-10, all of which employ a unique JAK-mediated intracellular signaling pathway [56,103,104]. Therefore, inhibition of JAK might be an effective strategy for treating CRS associated with COVID-19 infection [103,104].

Host chemokines are also potential targets of anti-CRS therapies [129]. Chemokines comprise a large group of small secreted proteins that generate signals through cell surface G protein-coupled chemokine receptors to stimulate cell migration. Chemokines are involved in all protective and destructive immune responses, with various chemokines produced during both infectious and inflammatory processes [130]. In addition to chemokines, other proteins and microbial products recruit leukocytes to infected or damaged tissues, where these cells participate in host inflammatory responses [131]. Meanwhile, chemokine receptors, considered easily controlled drug targets, are known to regulate inflammation and immune responses, prompting researchers to block interactions between chemokines and their receptors as an anti-inflammatory treatment strategy [129]. However, in practice, chemokine receptors have been difficult to antagonize, possibly reflecting the fact that such receptors engage in large numbers of diverse surface interactions with various chemokine ligands. Nevertheless, a few successful cases can be cited, such as that involving HIV co-receptors CCR5 and CXCR4, which were the first chemokine receptors targeted to block HIV entry into host cells [132,133]. Such early successes indicate that chemokine receptor antagonists show promise as targeted therapies for CRS [133,134]. Chemokine receptors may also serve as anti-CRS therapeutic targets based on their other functions, such as their regulation of host cell functional balance and functional stability. For example, although both dysfunctional macrophages and cytokines play essential roles in cytokine storm development, targeting cells may be more effective than targeting specific cytokines for achieving CRS control [135]. Nanomedicine-based macrophage-targeting therapies have already been shown to effectively reduce cytokine production in animal disease models by potentially regulating the balance between Tregs and Th17 cells to inhibit initiation and progression of inflammation [46,93].

As an additional thought, the association between gut microbiota and inflammation might serve as a target of anti-CRS treatments, since the results of many recent studies of gut bacteria suggest that imbalances of intestinal microflora may be root causes of many human diseases [136]. Fecal microflora transplantation can reverse intestinal microflora imbalances and alleviate gut inflammation, thus providing evidence in support of this concept. Treatments that regulate intestinal flora (e.g., probiotic treatments) may effectively control a variety of diseases, especially infection-triggered inflammatory diseases [137,138]. However, mechanisms by which various diseases harm the host differ, requiring the use of distinct intervention strategies to treat different diseases [136].

As a final thought, recent research on COVID-19 cytokine storms has highlighted potential clinical treatment options for cytokine storm-associated disorders in general. For example, vitamin D may alleviate cytokine storms by reducing levels of pro-inflammatory cytokines and increasing levels of anti-inflammatory cytokines, as suggested for the treatment of COVID-19-associated cytokine storms [36]. Moreover, therapeutic plasma exchange, which has also exhibited potential for use in treating severe COVID-19 cytokine storms, may act by effectively clearing inflammatory cytokines from the blood [105,106,107].

## 4. Discussion and Conclusions

In this review, we highlight differences between protective inflammatory responses and pathologic cytokine storms to help researchers design appropriate treatment strategies. Although inflammation and cytokine storms both involve host responses with or without infectious triggers, this review focuses on cytokine storms caused by infectious diseases. Infectious diseases that currently threaten human health around the world include influenza, bacterial sepsis, and other diseases that often cause sickness and death. During the progression of various infectious diseases, cytokine storms frequently occur that are generally treated by targeting both host and pathogen. However, more research is needed to improve treatment outcomes, Such as the effective treatment strategy for cytokine storms should also be considered chemokine storms [129]. Moreover, new therapies should not be based on achieving a single effect but should target and dampen inflammatory cascade reactions, alleviate cytokine storm-induced pathological damage, and balance the immune response to enable it to combat pathogen infection without triggering severe cytokine storm [13]. In addition, it is important to pay attention to differences in immune function and immune system characteristics between CRS-susceptible and resistant populations to formulate appropriate treatment plans for controlling CRS in different types of individuals. Although this paper mainly focused on CRS associated with infective microorganisms, the possibility of individual predisposition to CRS which is usually related to peculiar haplotypes or other conditions should not be ignored. For example, in a recent study, eight human leukocyte antigen (HLA)-B alleles were found to be associated with polymorphisms in three cytokines (IL-6, IL-10, and IL-12B) [139]. Another study analyzed the expression profiles of genes in monocytes from septic patients during compensatory anti-inflammatory response syndrome (CARS) and systemic inflammatory response syndrome (SIRS) [140]. The results showed that monocytes isolated from CARS patients exhibited decreased production of pro-inflammatory cytokines TNF-α and IL-6 but increased production of immunoregulatory cytokine IL-10 [140]. Ultimately, we predict that “multi-omics” immune system profiling will enable future clinicians to implement a series of effective therapeutic strategies to alleviate cytokine storms caused by various diseases [1].

In conclusion, cytokine storms caused by infectious diseases seriously affect the health of humans and susceptible animals, and often lead to multiple organ failures and death in patients. We believe that through an in-depth understanding of the pathological damage mechanism of cytokine storms, more effective and highly specific treatment strategies can be found in the future.

## Figures and Tables

**Table 1:** The incentive and related cytokines of CRS.

Incentive	Cytokines
CAR-T	IFN-γ, IL-2, IL-2Ra, IL-6, sIL-6R, GM-CSF, IL-1α, IL-1β, IL-10, IL-12, TNF-α, IFN-α, MCP-1, MIP-1α
H5N1	MCP-1, CXCL10, CXCL9, IL-8
H1N1	IL-8, IL-9, IL-17, IL-6, TNF-α, IL-15, IL-12p70
SARS	IL-1β, IL-6, IL-12, IFN-γ, IP10, MCP-1
MERS	IFN-γ, TNF-α, IL-15, IL-17
COVID-19	IL-2, IL-7, IL-10, G-SCF, IP10, MCP-1, MIP-1α, TNF-α

## Data Availability

Not applicable.
